# Supplementary material for: Investigating molecular crowding during cell division in budding yeast with FRET
Source: Curr Top Membr. Author manuscript; Available in PMC 2022 Jan 24. (PMC7612257; doi:10.1016/bs.ctm.2021.09.001)
Supplement: Supplementary Information [file EMS140802-supplement-Supplementary-Information.pdf]

Supplementary Information

Investigating molecular crowding during cell division in budding yeast with FRET

Sarah Lecinski[1,a], Jack W Shepherd[1,a,b], Lewis Frame[c], Imogen Hayton[b], Chris MacDonald[b], Mark C Leake[a,b]*

[1]These authors contributed equally

[a] Department of Physics, University of York, York, YO10 5DD

[b] Department of Biology, University of York, York, YO10 5DD

[c] School of Natural Sciences, University of York, York, YO10 5DD

* To whom correspondence should be addressed. Email mark.leake@york.ac.uk

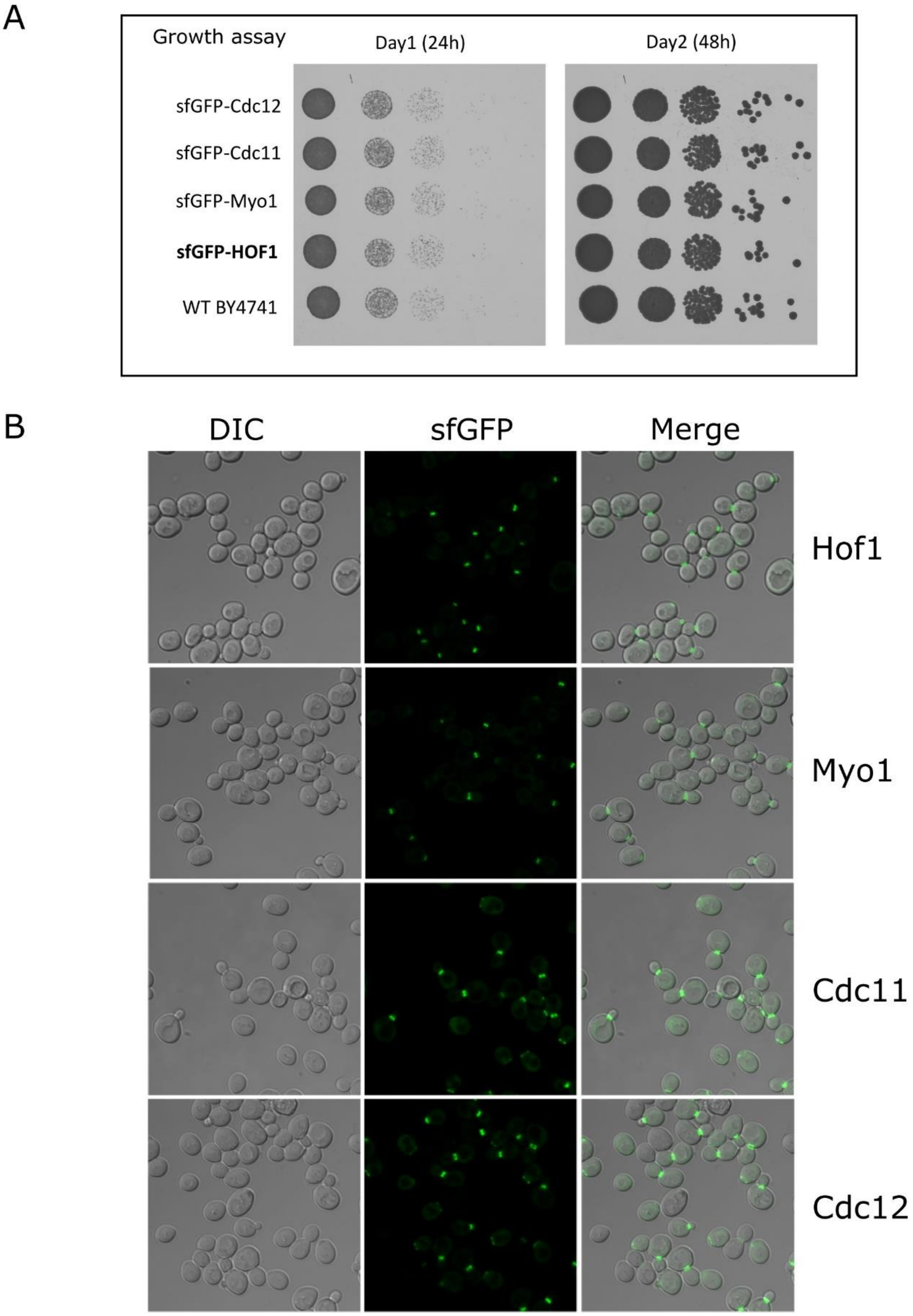

<u>Supplementary Figure S1: GFP-tagged mid-body markers do not affect growth</u>

A) Growth Assay for wild type BY4741 (WT) yeast and strains constitutively expressing indicated GFP tagged proteins under control of the *NOP1* promoter in BY4741 background strain. Cultures were

grown to mid-log phase, serially diluted 10-fold, plated on minimal media and growth recorded after 24 h and 48 h incubations at 30°C. Strains expressing a fluorescent markers (Hof1, Myo1, Cdc11 and Cdc12) grew similar to the WT strain with no growth defect to report for all strains tested.

B) Confocal imaging was used to localise each GFP-tagged strain, with DIC, GFP and merge micrographs included.

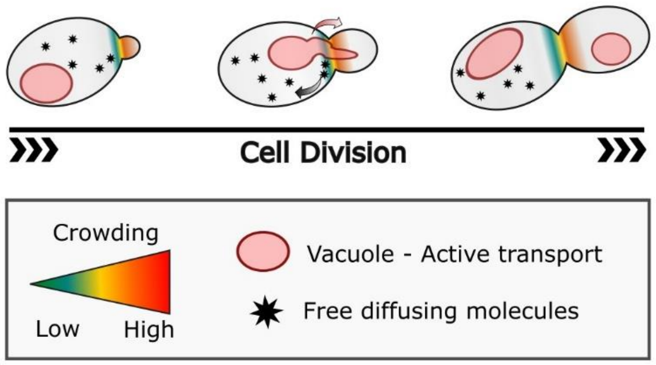

Supplementary Figure S2: Lateral diffusion barrier model for bud neck crowding.

Schematic diagram representing low to high crowding gradient from mother to daughter cells within the 200 nm region bordering the bud neck (Fig. 8). Hypothetic model for lateral diffusion barrier between the two connected cells depicted with colours, promoting the retention in the mother cell of free diffusion particles (black stars) while essential organelles such as the vacuole (in pink) can migrate to the daughter cell. In grey the rest of the cell where crowding profile of distribution not represented.

Supplemental statistic tables

The following tables shows all statistical tests performed for dataset presented and discussed in this study. All p values calculated using the non-parametric Brunner-Munzel test.

A) Comparison between the mother and daughter cell population, area and ratiometric FRET

**Comparison mother cells daughter cells**	Mother	Daughter
Mean area	14.453	5.124
Mean ratiometric FRET	0.347	0.344
Ratiometric FRET standard deviation	0.015	0.023
Mother-daughter area comparison p value	0	
Mother-daughter ratiometric FRET p value	0.03	

B) Daughter cell crowding and area dependency in budding yeast

**Daughter cell crowding and area dependency analysis**	Daughter with higher FRET than mother	Daughter with lower FRET than mother
Mean area	5.08	5.1
Median area	4.99	4.94
p value	0.87	

C) Local crowding at the bud neck - mother/daughter cell 200 nm region adjacent to the bud neck – three budding category stages (small: bud size smaller than 3µm^2 ; medium: bud size between 3µm^2 and 7µm^2 ; Large: bud size larger than 7µm^2)

**Axial analysis**	Small	Medium	Large
Daughter-bud comparison p value	0.05	2.46E-06	0.0002
Mother-bud comparison p value	6.82E-16	1.64E-42	7.66E-14
Daughter-mother comparison p value	7.8µE-14	1.61E-62	7.04E-30
Mean daughter ratiometric FRET	0.263	0.285	0.293
Mean bud neck ratiometric FRET	0.244	0.255	0.252
Mean mother ratiometric FRET	0.180	0.190	0.190
Daughter ratiometric FRET standard deviation	0.08	0.07	0.07
Bud neck FRET standard deviation	0.04	0.05	0.05
Mother cell ratiometric FRET standard deviation	0.05	0.04	0.05

D) Local crowding at the cell periphery

**At the cell periphery**	Outer-ring	All cell	Without outer-ring
Outer ring/all cell comparison p value	0.31		
All cell/cell without outer ring comparison p value		0.77	
Outer ring/cell without outer ring p value	0.24		
Mean Ratiometric FRET	2.197	2.215	2.22
Median Ratiometric FRET	2.184	2.204	2.208

E) Osmotic shock crowding in cells expressing the crGE sensor and labelled with FM4-64

**Combined CrGE & FM4-64 dye 0 to 1M NaCl shock**	Replicate 1	Replicate 2	Replicate 3
0 M/1 M comparison p value	5.60E-08	4.22E-06	0.0014
Mean ratiometric FRET shift (%)	4%	2.50%	1.50%
Median ratiometric FRET shift (%)	4.50%	3.10%	1.70%
